# Survival rate and use of revision components in total knee arthroplasty following unicompartmental knee arthroplasty or proximal tibial osteotomy: an analysis of 11,983 procedures from the Dutch Arthroplasty Register

**DOI:** 10.2340/17453674.2025.43333

**Published:** 2025-04-14

**Authors:** Maarten R HUIZINGA, Astrid J DE VRIES, Liza N VAN STEENBERGEN, Reinoud W BROUWER

**Affiliations:** 1 Department of Orthopedic Surgery, Martini Hospital, Groningen; 2 Dutch Arthroplasty Register (LROI), ’s-Hertogenbosch, The Netherlands

## Abstract

**Background and purpose:**

Unicompartmental osteoarthritis of the knee can be treated with high tibial osteotomy (HTO) or unicompartmental knee arthroplasty (UKA). This study aimed to investigate the use of revision components in total knee arthroplasty (TKA) after HTO or UKA and to investigate the revision-free survival of the 2 groups of TKA at different time points, including reasons for revision.

**Methods:**

TKAs after HTO and revision TKAs after failed UKA in the years 2007–2022 were selected from the Dutch Arthroplasty Register. For complexity, the usage of revision components was analyzed. Kaplan–Meier survival analysis was used to estimate survival rate. Multivariable Cox proportional hazards regression analyses were used to estimate the risk of revision, considering potential confounders. Indications of revision were evaluated.

**Results:**

11,983 procedures were analyzed (9,835 HTO-TKA and 2,148 UKA-TKA). In the HTO-TKA group, 3% had revision components in the TKA compared with 22% in the UKA-TKA group (P < 0.001). After 12 years’ follow-up the revision-free survival was 90.4% (95% confidence interval [CI] 89.6–91.2) for HTO-TKA and 81.7% (CI 79.3–84.1) for UKA-TKA. The multivariable Cox regression analysis showed that UKA-TKA was a significant risk factor for a revision of the TKA (hazard ratio 2.3, CI 1.8–2.6), compared with HTO-TKA. In both groups the most frequent reason for revision was instability, followed by patellar pain and loosening of the tibial component.

**Conclusion:**

TKAs after UKAs had higher use of revision components and a lower survival rate compared with TKAs following an HTO. The choice of surgical treatment should be considered for the young and active patient with unicompartmental knee osteoarthritis.

Patients with unicompartmental knee osteoarthritis can be treated non-surgically using an unloader brace or surgically with high tibial osteotomy (HTO) or unicompartmental knee arthroplasty (UKA) [1]. The choice between these 2 surgical options depends on patients’ characteristics, but also on surgeon preference [2-4]. Generally, there is a small group of patients who are suitable for both surgical procedures. Therefore only limited studies have been published comparing the HTO and the UKA [5-8].

Especially in younger patients a revision procedure to a total knee arthroplasty (TKA) can be expected after both surgical treatments. In the literature, survival rates differ from higher rates after 7–10 years from UKA compared with HTO (77% vs 60%) [4] to similar outcomes after 15–20 years [9]. The main reason for failure of HTO is progressive osteoarthritis and fallback of the correction osteotomy [10]. Reasons for failure of UKA include progressive symptomatic osteoarthritis in other compartments of the knee, aseptic/septic loosening, polyethylene wear, instability, and pain [11].

TKA after HTO or UKA might have different surgical challenges: 2 meta-analyses demonstrated that conversion to TKA after UKA required more revision components and thicker polyethylene inserts [12,13]. No differences in knee function scores including range of motion and postoperative complications between the 2 groups were demonstrated [12]. A recently published study found statistically significant higher Oxford Knee Scores in HTO-TKA compared with UKA-TKA, which were comparable to the Oxford Knee Scores after primary TKA [14].

The primary aim of this study was to investigate the use of revision components in TKA after HTO vs TKA after UKA. The secondary aim was to investigate the survival of the TKA, including reasons for revision.

## Methods

### Study design

This registry-based observational study adheres to the Strengthening the Reporting of Observational Studies in Epidemiology (STROBE) guidelines [15].

### Data source

The Dutch Arthroplasty Register (LROI) is a population-based registry containing information on all arthroplasties performed in the Netherlands (opt-out system). The LROI started in 2007 and achieved 100% coverage of all Dutch hospitals in 2012. The completeness of reporting of arthroplasties in the registry is high, with > 96% for primary TKAs since 2014 and > 93% for knee revision since 2014 [16,17]. Revision cause is not validated in the LROI. Several reasons for revision can be registered in the LROI. The LROI contains information on patient, surgery, and implant characteristics. Implant characteristics are cataloged in the LROI implant library, based on article numbers of prosthesis components [18].

### Data selection

Data from the LROI was obtained. 2 groups of procedures were selected: the first group contains TKAs after a high tibial osteotomy (HTO-TKA) with the TKA performed in the years 2007–2022. The LROI contains only the presence of a previous knee osteotomy as a binary variable (yes/no). Date, medial open vs lateral closed type, valgus vs varus direction, and femur vs tibia level of the osteotomy is not registered in the LROI. The type of knee osteotomy is not registered. However, based on clinical practice it is known that the vast majority are high tibia osteotomies (HTO). Therefore, we refer to all types of knee osteotomies as HTO. The second group contained UKAs (both medial and lateral) with a total revision to a TKA (UKA-TKA). For this group both procedures were performed in the years 2007–2022. All primary registered arthroplasties were performed for osteoarthritis in patients aged 18 years and older. Primary diagnosis for the osteotomies was not known and TKAs after UKA might had different indications for revision, i.e., instability, loosening, or fracture.

### Statistics

Descriptive statistics were used. Patient and surgical baseline characteristics are presented as numbers and percentages. Differences between the 2 groups were analyzed using a chi-square test.

The surgical complexity of the TKA is defined based on the components used. TKAs with primary/standard components were classified as low surgical complexity procedures, while TKAs with revision components were classified as high surgical complexity procedures. Revision components included every component added to a standard prosthesis, including stems, augments, and more constraint design than cruciate retaining or posterior stabilized. A chi-square test was used to analyze differences in the proportion of revision components between groups.

Prosthesis survival was estimated by Kaplan–Meier survival analysis with any revision as endpoint. Patient and surgery characteristics, including choice of implant design, were compared. Potential confounders (age, sex, ASA classification, component type, surgical year) were analyzed for their association with revision, which was estimated by a multivariable Cox proportional hazards regression analysis. These confounding variables were associated with either group (UKA-TKA or HTO-TKA) and outcome (survival of the TKA), based on the literature as well as clinical reasoning. Finally, indications for revision of the TKA were evaluated in both groups (multiple reasons for revision can be registered per revision).

A P-value below 0.05 is considered statistically significant. IBM SPSS version 25.0 (IBM Corp, Armonk, NY, USA) was used for the statistical analysis.

### Ethics, funding, data sharing, and disclosures

This study was evaluated by the Scientific Advisory Board of the LROI, which gave positive advice concerning the project. No approval by a local ethics committee was required. The authors did not receive any funding or have any conflict of interest regarding this article. Complete disclosure of interest forms according to ICMJE are available on the article page, doi: 10.2340/17453674.2025.43333

## Results

**Figure 1:**
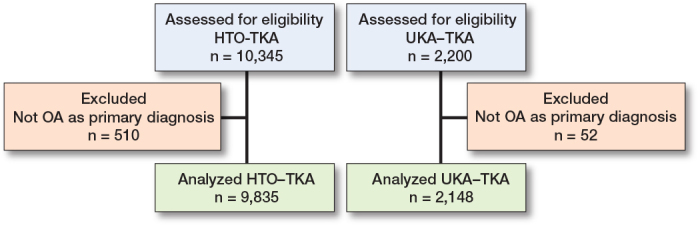
Flowchart of procedures in LROI dataset 2007–2022.

In the HTO-TKA group, 9,835 procedures and in the UKA-TKA 2,148 procedures were analyzed (Figure 1).

Patients in the HTO-TKA group are generally younger, are more often male, are less often obese (BMI > 30), more often ASA class I, and less often Charnley score A compared with the UKA-TKA group (Table 1).

**Table 1:** Baseline characteristics of the study population according to group at the time of TKA surgery. Values are count (%) unless otherwise specified

Factor	HTO-TKA	UKA-TKA	P value
Patient characteristics			
n		9,835 (	2,148 (
Age, mean (SD)	63.1 (9.0)	64.6 (8.9)	< 0.001
Age groups			< 0.001
< 50	594 (6.0)	68 (3.2)	
50–59	2,889 (29)	582 (27)	
60–69	3,965 (40)	849 (40)	
70–79	2,044 (21)	544 (25)	
≥ 80	342 (3.5)	105 (4.9)	
Sex			< 0.001
Male	5,473 (56)	728 (34)	
Female	4.354 (44)	1.399 (66)	
BMI ^a^, mean (SD)	29.3 (4.7)	29.7 (4.6)	0.009
BMI groups ^a^			0.003
< 18.5	10 (0.2)	1 (0)	
> 18.5–25	1,045 (17)	277 (16)	
> 25–30	2,767 (45)	713 (41)	
> 30–40	2,241 (37)	714 (41)	
> 40	144 (2.3)	33 (1.9)	
Smoking ^a^			0.4
Yes	761 (13)	202 (12)	
No	5,270 (87)	1,512 (88)	
ASA class			< 0.001
I	2,151 (22)	321 (15)	
II	6,152 (64)	1,423 (67)	
III–IV	1,341 (14)	384 (18)	
Charnley score ^a^			< 0.001
A	2,745 (44)	857 (55)	
B1	2,089 (33)	297 (19)	
B2	1,232 (20)	335 (21)	
C	177 (2.8)	78 (5.0)	
Surgical characteristics			
Surgical year,			
median (IQR)	2015 (7)	2017 (6)	< 0.001
Type of components (proxy for surgical complexity)			< 0.001
Primary component	9,385 (97)	1,621 (78)	
Revision component	268 (2.8)	458 (22)	
Prosthesis type			< 0.001
Posterior stabilized	5,816 (61)	1,417 (69)	
Cruciate retaining	3,654 (38)	556 (27)	
High constraint	53 (0.6)	63 (3.1)	
Hinged	31 (0.3)	15 (0.7)	
Fixation			< 0.001
Cemented	9,019 (92)	1,966 (92)	
Cementless	414 (4.2)	35 (1.6)	
Hybrid	344 (3.5)	138 (6.5)	

Note: Numbers do not add up to total due to missing values.

a The variables BMI, smoking, and Charnley score were registered since 2014.

In the HTO-TKA group the proportion of TKAs with revision components was much lower than in the UKA-TKA group: 3% vs 22% (P < 0.001). In the UKA-TKA group more posterior stabilized and hinged TKAs were used (Table 1).

**Figure 2:**
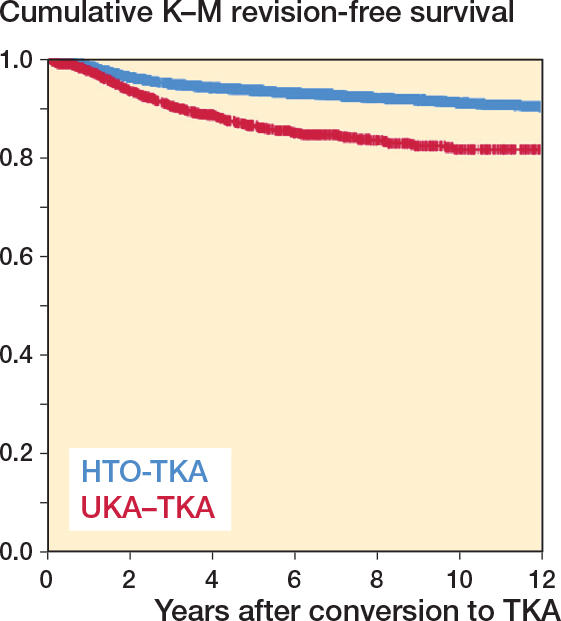
Cumulative Kaplan–Meier revision-free survival of the TKA in the HTO-TKA and UKA-TKA groups until 12 years’ follow-up.

The mean follow-up in the HTO-TKA group was 6.9 (SD 4.2) years and in the UKA-TKA group 4.8 (SD 3.5) years. The Kaplan–Meier survival curve showed a higher survival rate for HTO-TKAs compared with UKA-TKAs for the entire follow-up period (Figure 2, Table 2).

**Table 2:** Revision-free survival rates and patients at risk of revision at different time points in the HTO-TKA and UKA-TKA groups

Follow-up, years	HTO-TKA		UKA-TKA	
	% (CI)	n at risk	% (CI)	n at risk
1	98.5 (98.3–98.7)	8,996	97.5 (96.8–98.2)	1,853
3	94.9 (94.4–95.4)	7,631	90.3 (88.9–91.7)	1,307
5	93.6 (93.1–94.2)	6,110	86.5 (84.8–88.2)	887
10	91.1 (90.4–91.8)	2,574	81.7 (79.3–84.1)	219
12	90.4 (89.6–91.2)	1,426	81.7 (79.3–84.1)	78

CI = 95% confidence interval.

The multivariable Cox regression analysis showed that the UKA-TKA group had statistically significant higher risk of revision than the HTO-TKA group (hazard ratio [HR] 2.2, CI 1.8–2.6), when adjusted for potential confounders (Table 3). The confounders male sex, being of younger age, ASA classification III–IV, need for revision components, and a less recent surgical year increased the risk of revision after the TKA.

**Table 3:** Adjusted HR with 95% confidence interval for risk of revision by type of primary procedure

Factor	HR (CI)	P value
Group		
HTO-TKA	1.00 (reference)	
UKA-TKA	2.17 (1.85–2.55)	< 0.001
Sex		
Male	1.00 (reference)	
Female	0.85 (0.75–0.97)	0.02
ASA		
I	1.00 (reference)	
II	1.05 (0.90–1.24)	0.5
III–IV	1.28 (1.02–1.63)	0.03
Age		
< 50	2.76 (2.22–3.45)	< 0.001
50–59	1.63 (1.40–1.90)	< 0.001
60–69	1.00 (reference)	
70–79	0.61 (0.49–0.77)	< 0.001
≥ 80	0.40 (0.22–0.73)	0.003
Component type		
Primary	1.00 (reference)	
Revision	1.52 (1.20–1.92)	0.001
Year of TKA procedure	0.98 (0.96–0.99)	0.01

HR = hazard ratio; CI = 95% confidence interval.

Year of TKA procedure ranged between 2007 and 2022, included in Cox regression analysis as a continuous variable.

In both groups the most frequent reason for revision of the TKA was instability, followed by patellar pain and loosening of the tibial component (Table 4).

**Table 4:** Reasons for revision of the TKA surgery until 12 years follow-up that were registered in both groups

Factor	HTO-TKA	UKA-TKA
Number of revisions	n = 692	n = 250
Reasons for revision (%)		
Instability	33	32
Patellar pain	25	28
Loosening of tibial component	22	27
Infection	18	14
Malalignment	15	16
Loosening of femoral component	7.2	4.0
Arthrofibrosis (since 2014)	4.5	6.0
Wear of inlay	4.5	2.8
Patellar dislocation	1.9	0.8
Periprosthetic fracture	1.3	0.4
Loosening of patellar component	1.2	2.8
Progression of OA	1.0	2.4
Other	8.4	9.2

Note: numbers do not add up to 100% per group because multiple reasons for revision can be registered. In 5 records (4 in HTO-TKA and 1 in UKA-TKA group) the reason for revision was missing.

## Discussion

We aimed to investigate the surgical complexity of TKA after HTO vs TKA after UKA and to investigate the survival of both groups at different time points, including reasons for revision. We found that the use of revision components was significantly higher in TKAs after HTO compared with TKAs after UKA in the Dutch register. UKA-TKA showed a significantly lower survival rate at 10 years’ follow-up compared with HTO-TKA. UKA-TKA was a significant risk factor for revision compared with HTO-TKA.

The meta-analysis by Lee et al. showed, in agreement with our results, that UKA-TKA conversion required significantly more revision components and thicker polyethylene inserts than HTO-TKA conversion [12]. These differences are probably, at least to some extent, related to the difference in indications for revision to a TKA. The results of the current study are also confirmed by Van Raaij et al., who showed that HTO-TKA conversion was technically more challenging than a regular TKA, but in all cases a standard primary total knee prosthesis could be used [19]. A study from the New Zealand Joint Registry found that more revision components were used in the UKA-TKA group (28.3%) compared with the HTO-TKA group (2.4%) [20]. The proportion of revision components in the UKA-TKA group in that study was even higher than in our study, with similar criteria for use of revision components. In contrast to our results, they found no significant difference in TKA revision rate between the 2 groups (no adjustments for patient or surgical characteristics were included in their analysis) [20].

A recent registry study showed a similar revision rate for HTO-TKA and UKA-TKA while both risks were higher than in a primary TKA [14]. In the study of Lee et al., TKA after HTO showed a lower revision rate than TKA after UKA (OR 0.65, 95% confidence interval [CI] 0.55–0.78) [12].

In a Swedish register study, a significant higher risk of revision was seen in UKA-TKA and TKA after closed-wedge osteotomy, but no significant difference in TKA after open-wedge osteotomy [21]. A Danish register study including a propensity-score weighted analysis did confirm our findings, showing a lower 5-year survival of 88% in the UKA-TKA group compared with 94% in the HTO-TKA group (our data showed a 5-year survival of 87% and 94%, respectively). They concluded that the TKA revision risk was more than doubled by a previous UKA compared with a previous HTO in accordance with our study [22]. A meta-analysis by Shen et al. showed similar results regarding the use of revision components and also a higher revision rate of UKA-TKA, probably due to the complexity of the surgical procedure [13].

Considering the reasons for revision of the TKA, we found that patellofemoral pain, instability, and loosening of the tibial component were the most frequently reported in both groups. Pearse et al. found infections to be the most frequent cause in both groups, followed by patellofemoral pain, instability, and loosening of components [20]. Relatedly, it should be noted that anterior knee pain can have various causes [23]. A possible explanation for the discrepancy in reasons for revision might be that our results are based on more than 1 reason for revision, which might have resulted in different outcomes compared with systems where a hierarchical order is used. Other studies did not look at the reasons for revision of the TKA [12,14,22].

### Strengths

A major strength of this study is the population-based nature of this registry study. The LROI has a completeness of over 97% [16,17]. Furthermore, this large observational cohort provides the possibility to perform adjusted analyses, where differences between groups can to some degree be corrected for in the analysis.

### Limitations

The UKA-TKA group contains several unfavorable demographic variables compared with HTO-TKA group, which may influence revision rate of the TKA. Even though the regression analysis is identifying UKA as a single risk factor, it is important to consider the association of confounders in the analysis. There might also be some residual confounding from unmeasured variables (e.g., patient expectations, surgeon experience/preference, joint/bone quality). The thickness of the insert has not been analyzed, as this data was not available. If the thickness of polyethylene inserts had been included in the analysis, the difference in complexity would have been even greater in disadvantage of the UKA-TKA group, as by using a thicker insert to compensate for bone loss augments and stems may be avoided [24]. Moreover, date of osteotomy, type of osteotomy (open vs closed), direction (valgus vs varus), and level (proximal tibia vs distal femur) are unknown in the LROI register. Apart from survival rates, patient-reported outcome measures are important outcome measures.

### Conclusion

TKAs after UKAs had a higher use of revision components and a lower survival rate than TKAs following a HTO. This is important to consider for the young and active patient in need of knee surgery for unicompartmental knee osteoarthritis.

## Notes

RB, AdV, and LS contributed to the conception of this study. MH collected data and drafted the manuscript. AdV conducted the statistical analysis. RB, LS, and AdV revised the manuscript. All authors approved the final manuscript.

Handling co-editor: Per Henrik Randsborg

*Acta* thanks Eirik Aunan, Andrea Baldini, and Annette W-Dahl for help with peer review of this manuscript.
